# Postmastectomy Breast Reconstruction in Irradiated Patients: A 12-year follow-up of Deep Inferior Epigastric Perforator and Latissimus Dorsi Flap Outcomes

**DOI:** 10.1016/j.jpra.2024.10.008

**Published:** 2024-10-18

**Authors:** Åsa Edsander-Nord, Armin Assareh, Martin Halle, Ann-Charlott Docherty Skogh

**Affiliations:** a Department of Plastic surgery and Craniofacial surgery, Karolinska University hospital, Stockholm, Sweden; b Department of Molecular Medicine and Surgery, Karolinska Institutet, Stockholm, Sweden; c Akademikliniken, Gothenburg, Sweden; d Department of Clinical Science and Education, Karolinska Institutet, Södersjukhuset, Stockholm, Sweden

**Keywords:** Breast reconstruction, Irradiation, DIEP, LD, Patient satisfaction, Follow-up

## Abstract

The aim of the current study was to conduct a 12-year follow-up on the authors´ previously evaluated group of irradiated patients who underwent postmastectomy breast reconstruction with deep inferior epigastric perforator (DIEP) and latissimus dorsi (LD)-flaps with implant.

The follow-up involved 67% of the patients from the original cohort (17 DIEP and 13 LD). Patient-reported outcome measures (PROMS) were measured using the BREAST-Q, SF-36, a satisfaction form, and Disabilities of the Arm, Shoulder, and Hand (DASH) questionnaires. Aesthetics were evaluated by a board of independent plastic surgeons.

The average follow-up time was 12.6 years (DIEP) and 11.8 years (LD). Contralateral symmetry procedures were performed on 15 DIEP and 9 LD patients. Both groups underwent an average of 2.5 reconstructive procedures. The BREAST-Q and satisfaction questionnaires showed no group difference. SF-36 showed no group difference but had significantly higher values in both groups compared to the general population with regard to the physical role limitations (p=0.034 and p=0.004, respectively). The DASH scores showed minimal shoulder function impact in the LD group.

Aesthetic evaluations revealed a discrepancy between the opinions of the patients and surgeons, with patients valuing the size (p=0.015) and overall aesthetic (p=0.012) of the reconstructed breast higher in the DIEP group. The weighted kappa analysis showed poor agreement between patients and surgeons. Over time, the patients´ preferences shifted from LD to DIEP flaps, possibly due to the more natural aging process associated with autologous reconstruction. This underscores the importance of long term follow-up studies.

## Introduction

Breast reconstruction is an important part of breast cancer treatment and plays a major role in improving health-related quality of life after mastectomy. As the overall survival rate is improving in patients with breast cancer and has now reached 90% five-year survival in Sweden, the importance of body image and quality of life has also increased. Autologous tissue transfer is often performed to achieve improved tissue quality during breast reconstruction after radiotherapy. In the last few years, several studies have investigated the long-term results from different viewpoints.^1^^,^^2^

The authors previously evaluated the same cohort of irradiated patients with regards to postmastectomy breast reconstruction with deep inferior epigastric perforator (DIEP) and latissimus dorsi (LD) flaps.^3^ The original study was conducted 3 years after the breast reconstruction and published in 2012, and one of the findings was a difference in the opinions of patients and plastic surgeons regarding the outcomes. Since then, the use of BREAST-Q has become the gold standard in patient-reported outcome measures (PROMs) in breast reconstruction patients.^4^^,^^5^

The aim of this study was to conduct a 12-year follow-up of PROMs and aesthetic results, study the impact on shoulder function in the LD group, and evaluate the difference between the opinions of patients and plastic surgeons regarding the outcomes over time. All patients underwent reconstruction with either a DIEP or LD flap with an implant.

## Patients and Methods

The study was approved by the regional ethical committee (No 2017/1462-31).

**Figure 1:**
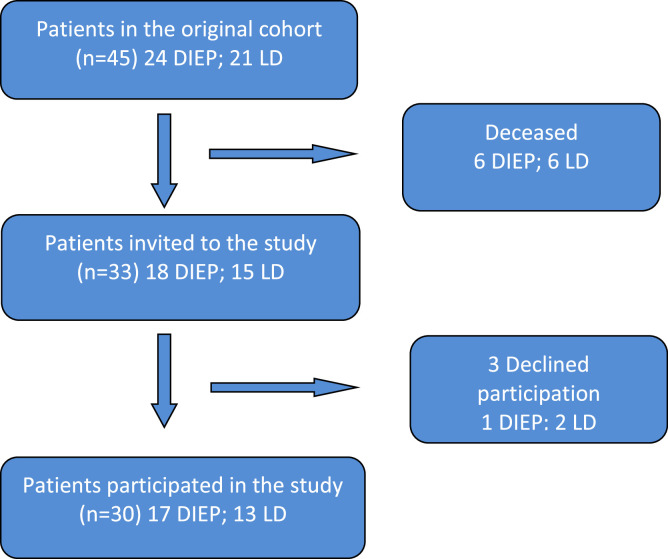
STROBE flow chart of the study

The original study included 24 DIEP flap and 21 LD flap patients. All of them previously underwent mastectomy, irradiation of the chest wall, and received secondary breast reconstruction between 2004 and 2006 at the Karolinska University hospital, where autologous reconstructions during that period were performed on irradiated patients in a delayed setting. All the patients had nipple reconstruction with modified star flaps and tattooing of the areolae. At the follow-up, 12 patients had died (6 DIEP and 6 LD flap patients). The remaining 33 patients were contacted via mail with an invitation to participate in the study (Figure 1). All patients were asked to complete the original study-specific questionnaire, the BREAST-Q reconstruction module and the Short Form Health Survey (SF-36). The study-specific questionnaire modified from Brandberg et al^6^, ^7^, ^8^ considered the aesthetic result and emotional consequences of the reconstruction. The LD flap patients were also asked to complete the Disabilities of the Arm, Shoulder, and Hand (DASH) questionnaire and the BREAST-Q latissimus dorsi module. All patients were also contacted via telephone and invited to the outpatient clinic to have photographs taken of their breasts and donor site. The patients were photographed in four standardized positions, and the patients who underwent reconstruction with a LD flap also had a fifth photo taken of the donor site. A blinded expert panel evaluated the aesthetic outcome in the photographs using the same seven items in the study-specific questionnaire concerning the aesthetic result as reported by the patients. The panel consisted of three plastic surgeons who had not treated the patients. The reported answers were further compared for concordance between patients and the panel.

### Statistical Analysis

Statistical analysis was performed using the unpaired t-test for continuous variables, the study-specific questionnaire^6^, ^7^, ^8^ and subjective aesthetic evaluation of the photographs. Mann-Whitney U test was used for evaluation of the SF-36 and BREAST-Q results. The agreement between the members of the expert panel and patients was measured using weighted kappa measurement. The weighted kappa measurement evaluates the concordance of the reported answers from two or more evaluators. Significant differences were defined by values of p<0.05. The software used was GraphPad Prism by Dotmatics and SPSS Statistics.

## Results

Demographic data are listed in Table 1. During the study, the mean age of the patients was 62 years in both groups. The mean follow-up time was 12.6 years in the DIEP group and 11.8 years in the LD group. There was no difference between the groups regarding time from radiotherapy to reconstruction or time from radiotherapy to follow-up. One DIEP flap patient and 1 LD patient underwent bilateral reconstruction during the primary surgery (Table 2). Two additional DIEP flap patients and 1 additional LD patient had implant-based contralateral reconstructions during the follow-up period due to contralateral cancer or prophylactic surgery. One LD patient had a DIEP flap reconstruction before the study.

**Table 1:** Demographic Data.

Characteristics	DIEP flap patients	LD flap patients
No. of patients	17	13
Mean age at reconstruction, yr	49	50
Mean age at follow-up, yr	62	62
Mean follow-up time, yr	12.6	11.8
Time from XRT to reconstruction, yr	2.8	3.5
Time from XRT to follow-up, yr	15.4	15.3
Mean BMI, kg/m^2^	25.5	24.2
Smokers	2	1
Hypertension	0	2
Previous thromboembolism	0	0
Diabetes mellitus	0	0
ASA 1	14	11
ASA 2	3	2
ASA 3	0	0

LD, latissimus dorsi; XRT, external radiotherapy; BMI, body mass index; ASA, American Society of Anesthesiologists classification; DIEP, deep inferior epigastric perforator

**Table 2:** Reconstructive Procedures.

	DIEP flap	LD flap
No. of flaps	18	13
Bilateral reconstructions	3 (1 bilateral DIEP)	3 (1 bilateral LD)
Delayed reconstruction	18 (1 primary and delayed)	13
Reconstructive procedures to achieve final result		
Mean	2.4	2.5
Range	2-5	1-4
Symmetry procedures	15/18 (83%)	9/13 (69%)
Reduction	9	5
Mastopexy	4	1
Implant change	0*	5
Liposuction	3	1

⁎ No DIEP flap patients required additional implants. DIEP, deep inferior epigastric perforator; LD, latissimus dorsi

Fifteen of the 18 DIEP flap patients and 9 of the 13 LD flap patients underwent contralateral symmetry procedures, an increase of 8% and 17%, respectively, during the 8 years since the previous study. The total number of reconstructive procedures is shown in Table 2. The 5 implant exchanges in the LD flap and implant patients that occurred during the follow-up was due to capsular contracture and was aimed at improving the shape and size symmetry.

There was no significant difference between the breast reconstruction groups regarding the eight domains of the SF-36 form (Table 3). Compared to a reference population of Swedish women aged 65-69 years,^9^ there was a difference in role physical, where both reconstructive groups have significantly higher values compared to the reference population (DIEP, p=0.034 and LD, p=0.004). In role emotional, there was a significantly higher score in the LD group compared to the reference population as well (p=0.03).

**Table 3:** Short Form-36 DIEP vs. LD and Swedish women aged 65-69 years from the general population.

	DIEP Mean (SD)	LD Mean (SD)	p*	Ref Population	DIEP–ref *p*	LD-ref *p*
No. of patients	17	13		1000		
**Physical functioning**	74.4 (23.2)	81.5 (19.7)	0.384 (n.s)	68.1 (25.7)	0.316 (n.s)	0.061 (n.s)
Physical role limitations	73.2 (31.3)	85.6 (18.7)	0.217 (n.s)	50.7 (43.4)	***0.034***	***0.004***
Body pain	67.7 (25.7)	71.0 (25.9)	0.731 (n.s)	63.4 (28.1)	0.531 (n.s)	0.332 (n.s)
General health perceptions	59.2 (28.2)	67.3 (20.0)	0.387 (n.s)	56.9 (23.8)	0.694 (n.s)	0.117 (n.s)
Energy/Vitality	57.7 (26.9)	64.4 (20.4)	0.461 (n.s)	53.2 (23.2)	0.429 (n.s)	0.084 (n.s)
Social functioning	80.9 (30.0)	87.5 (23.0)	0.516 (n.s)	75.5 (29.0)	0.447 (n.s)	0.138 (n.s)
Emotional role limitations	78.4 (31.4)	87.2 (20.0)	0.386 (n.s)	61.0 (43.4)	0.100 (n.s)	***0.030***
Mental health	72.9 (22.1)	75.4 (22.5)	0.763 (n.s)	67.8 (21.1)	0.324 (n.s)	0.198 (n.s)

n.s, not significant; LD, latissimus dorsi; DIEP, deep inferior epigastric perforator

The analysis of the questions regarding aesthetic outcomes^3^^,^^10^ in the two groups showed no significant difference in the shape of the breast, symmetry between the breasts, scar on the reconstructed breast, donor site, or in nipple-areola reconstruction. However, there was a difference in opinion regarding the size of the reconstructed breast between the patients and surgeons, where the patients valued the DIEP reconstructed breast higher (p=0.015). There was also a significant difference regarding the overall aesthetic result, where the patients were more pleased with the DIEP flaps than the surgeons (p=0.012) (Table 4).

**Table 4:** Questions regarding the Aesthetic Outcome.

	Patients´ opinions			Plastic Surgeons´ opinions			Patients´ vs. Plastic surgeons (*p*)	
	DIEP Mean (SD)	LD Mean (SD)	*p*	DIEP Mean (SD)	LD Mean (SD)	*p*	DIEP	LD
No. of patients	17	13		12	10			
Size of the breast	6.1 (1.4)	5.5 (1.9)	0.327	4.8 (1.2)	4.2 (1.4)	0.292	***0.015***	0.084
Shape of the breast	5.6 (1.5)	5.1 (1.8)	0.414	5.0 (1.3)	4.3 (1.5)	0.254	0.273	0.270
Symmetry between the breasts	4.8 (1.9)	4.1 (2.4)	0.380	4.2 (1.3)	3.7 (1.6)	0.428	0.352	0.654
Softness of the breast	5.5 (1.7)	4.2 (1.9)	0.058					
Scar on the reconstructed breast	5.1 (1.8)	4.2 (2.2)	0.228	5.2 (1.2)	4.5 (1.3)	0.204	0.868	0.706
Sensibility of the breast	3.9 (2.2)	3.3 (2.1)	0.457					
Nipple-areola complex	4.2 (2.0)	2.8 (1.9)	0.062	4.8 (1.4)	4.2 (1.2)	0.299	0.379	0.055
Scar on donor site	4.6 (2.0)	4.7 (1.9)	0.890	5.0 (1.3)	5.4 (1.3)	0.481	0.549	0.330
Problem/bulging on the abdomen	4.2 (1.9)							
Problem from donor site on the back		5.2 (2.2)						
Overall aesthetic result	6.1 (1.5)	5.3 (2.0)	0.220	4.8 (0.9)	4.1 (1.4)	0.171	***0.012***	0.122

LD, latissimus dorsi; DIEP, deep inferior epigastric perforator; SD, standard deviation

Analysis of the Breast-Q results shows no differences between the two groups (Table 5).

**Table 5:** Breast-Q DIEP vs LD.

	DIEP Mean (SD)	LD Mean (SD)	p*
No. of patients	17	13	
Satisfaction with breasts	68.6 (18.2)	62.8 (21.2)	n.s
Psychosocial wellbeing	73.5 (22.8)	79.2 (17.4)	n.s
Physical wellbeing breasts	87.3 (20.7)	85.7 (14.2)	n.s
Physical wellbeing abdomen	77.6 (16.4)		-
Satisfaction with abdomen-Appearance	2.9 (0.9)		-
Umbilicus	3.0 (0.9)		
Scars	2.9 (0.9)		
Nipple-areola complex	2.6 (0.9)	2.1 (1.0)	n.s
Sexual wellbeing	52.6 (16.8)	51.1 (16.5)	n.s
Satisfaction with preoperative information	71.6 (20.7)	71.8 (21.6)	n.s
Satisfaction with surgeon	88.6 (16.3)	79.1 (20.7)	n.s
Satisfaction with medical staff	97.4 (7.7)	95.5 (11.2)	n.s
Satisfaction with admin staff	92.9 (14.8)	93.2 (13.7)	n.s
Satisfaction with back		75.6 (25.4)	-
Satisfaction with shoulder function		58.9 (19.9)	-

⁎ *p-value calculated using the Mann-Whitney U test*. n.s, not significant; LD, latissimus dorsi; DIEP, deep inferior epigastric perforator

The responses of the LD group to the DASH questionnaire showed little effect on the shoulder function with a mean value of 11.5 (Table 6), where a DASH value between 20 and 30 indicates a level of function enabling work. A level of 50-60 indicates the inability to work and activities of daily living may be impaired.^11^

**Table 6:** DASH score for LD flap. A lower value indicates fewer symptoms.

Activity	LD flap (mean)
Activity/symptom (0-100)	11.5 (n=13)
Work	1.6 (n=8)
Music/exercise	28.8 (n=5)

LD, latissimus dorsi; DASH, Disabilities of the Arm, Shoulder, and Hand

The weighted kappa values showed good to moderate agreement between the surgeons regarding the results in general, whereas the agreement between the surgeons and patients regarding the results remained poor (Table 7).

**Table 7:** Weighted Kappa Values.

	Between Plastic surgeons			Between Plastic Surgeons and Patients		
	Mean	Range	Grade	Mean	Range	Grade
Size of the breast	0.47	0.34-0.66	Moderate	0.14	0.05-0.25	Poor
Shape of the breast	0.41	0.17-0.61	Moderate	0.18	0.08-0.25	Poor
Symmetry between the breasts	0.61	0.42-0.79	Good	0.15	0.11-0.20	Poor
Scar on the reconstructed breast	0.26	−0.12-0.59	Fair	0.06	−0.04-0.14	Poor
Nipple-areola complex	0.53	0.48-0.57	Moderate	0.08	0.01-0.14	Poor
Scar on donor site	0.43	0.20-0.60	Moderate	0.25	0.13-0.37	Fair
Overall aesthetic result	0.50	0.29-0.78	Moderate	0.19	0.13-0.22	Poor

Key: ≤0.20, poor; 0.21-0.40, fair; 0.41-0.60, moderate; 0.61-0.80, good; and 0.81-1.00, very good.

## Discussion

The current study highlights the importance of long term follow-up of our previous study of postmastectomy breast reconstruction in irradiated patients, evaluating the satisfaction after reconstruction with either DIEP or LD flaps and implant.^3^ The original study was conducted 3 years after the breast reconstruction and published in 2012, showing a difference between the opinions of the patients and plastic surgeons regarding the outcomes. The patients were more satisfied with LD flap reconstruction, which may be related to the donor-site scar. However, the surgeons favored DIEP flap reconstruction with regards to the size and shape of the breast.

In the present study, we performed a long term follow-up of the same patient group 12 years after the surgery. The number of symmetry procedures was higher in the DIEP group, 15/17 compared to 9/13 in the LD group. The observed increase in symmetry surgeries within the DIEP group may be attributable to a combination of patient selection factors and surgical capabilities. Patients selected for DIEP flap reconstruction often present with larger and more ptotic breasts in combination with the preferred abdomen for DIEP. This demographic is frequently motivated by aesthetic outcomes, leading to a higher likelihood of pursuing symmetry surgery. In contrast, patients opting for LD flap reconstruction are typically slimmer, often resulting in smaller breast volume and less sagging, which may reduce the perceived need for symmetry correction. Contralateral implants are not offered for the contralateral breast. This highlights the importance of tailoring reconstructive approaches to individual patient anatomy and aesthetic goals, ensuring optimal outcomes in breast reconstruction.

SF-36 showed that both groups felt better than the reference population in their physical function, and the LD group felt more vital and well-functioning emotionally. There is no difference in opinions between the groups regarding the aesthetic outcome of breast reconstruction, and no differences were observed in the Breast-Q results. DASH scores regarding shoulder function showed little effect of the surgery in the LD group after 12 years, which is in concordance with a study by Garusi et al.^12^

Similar to the previous study, the difference between the patients´ and surgeons´ opinions about the result remained, but the patients appreciated the DIEP reconstruction more than the surgeons did, in terms of the size of the breast and overall aesthetic result after 12 years. The scores regarding the aesthetic outcome showed a trend toward higher values in the DIEP group over time, indicating a positive change with aging. In the LD group, there is a trend toward lower scores over time (Table 4), possibly indicating an increasing side difference, due to capsular contracture, between the breasts related to implant reconstruction in combination with the LD flap. The donor sites complications appeared to diminish in both groups over time, but overall there is no significant difference over time regarding the aesthetic outcome, except for a difference in the LD group regarding the nipple-areola reconstruction.

Löfstrand et al.^13^ studied donor-site satisfaction in DIEP and LD flaps and found that impairment due to muscular weakness of the donor site was more common in the LD group than that in the DIEP group. Bulging was common in the DIEP group and increased over time. Regarding the aesthetic appearance of the donor site, the patients in the DIEP group were less satisfied than those the LD group.^2^ This is in accordance with the results of our first study, but not with the results in the long term follow-up. In a comparison of patient-reported outcomes measured using BREAST-Q by Löfstrand et al, DIEP reconstruction was found to be superior to LD reconstruction after 7 years,^14^ which is also indicated in our present study.

In a study by Demiri et al, the outcome and characteristics of the patients undergoing secondary breast reconstruction with fat augmented LD flaps (FALD) and DIEP flaps were studied.^15^ The patients in the FALD group were thinner, with smaller breast size, and the satisfaction with the result showed no significant difference, but there was a tendency toward higher scores in the DIEP group. The follow-up time was 12 months with no record of donor-site morbidity. When using fat augmentation instead of an implant, there is a possibility that the long-term outcome with LD flaps improves, but this remains to be investigated.

A study by Petrou et al tried to identify the ideal breast reconstruction method after mastectomy in a mixed group of primary and delayed reconstructions comparing implant-based reconstruction, DIEP and LD flaps with or without implants. The authors concluded that DIEP reconstruction appeared to be the most satisfactory and best experienced reconstruction method for patients, among radiated and nonradiated patients. They noted that good results were reported overall for all breast reconstruction procedures, with more reserved scores for LD.^16^ As radiotherapy has such a great impact on the reconstructive result, their conclusions might not be valid in a homogenous group of patients with postoperative radiotherapy treatment.^17^

The results of a longitudinal multicenter prospective cohort study investigating complication rates of different types of breast reconstruction after mastectomy was presented by Bennett et al.^18^ Overall, complications were noted in 32.9% of the patients, with reoperative complications in 19.3% and wound infections in 9.8% of the patients. Two years postoperatively, patients undergoing any autologous reconstruction type, including DIEP and LD flaps, had significantly higher odds of developing any complication compared with those undergoing expander or implant reconstructions. LD flaps had lower risk of reoperative complications similar to expander or implant reconstructions, and expander or implant and direct to implant had higher failure rates (7.1%).

In a randomized controlled trial (RCT) by Brorson et al.^19^ regarding patient-reported outcome and quality of life 7 to 8 years after secondary breast reconstruction, no distinct differences in long-term health-related quality of life could be seen for the different methods (LD or DIEP flap in the radiated arm and a thoracodorsal flap and implant or an expander in the nonradiated arm). There was a clear improvement in health-related quality of life compared to prereconstruction in all groups, but the effect of specific reconstructive methods on the scores could not be reliably demonstrated.

Leonardis et al examined the influence of different breast reconstructive methods on shoulder biomechanics. LD flap plus subpectoral implant patients were found to exhibit reduced shoulder strength and stiffness compared with subpectoral implant and DIEP flap patients.^20^ They found that objective measures of shoulder biomechanics were predictive of patient-reported physical and psychosocial well-being, and emphasized the need for improved perioperative screening for shoulder functional deficits in patients undergoing breast reconstruction.

The effects on shoulder function and also on spinal posture play a role in the postoperative period for patients who underwent breast reconstruction with LD flaps. Kim et al have demonstrated that unilateral LD muscle flap harvest for breast reconstruction might be associated with changes in spinal posture in the long term, especially in older patients. Multivariable analyses revealed that use of the LD flap was associated with a significantly increased rate of postoperative scoliosis, but no association between the use of LD muscle flap and development of back pain was observed.^21^ In a recent study by Löfstrand et al.,^14^ they reported a lower satisfaction with back and shoulder function in patients reconstructed with LD flaps compared to DIEP flaps. They concluded that delayed LD reconstruction should be used with care, especially in older patients who have undergone axillary surgery and axillary radiotherapy.

The patients reconstructed with LD flaps in our study reported little effect on the shoulder function, but the number of patients was small, and a larger study of LD reconstructions might be of value, where radiological examination of the spinal posture is done and compared to clinical symptoms.

### Limitations of the Study

This study has some limitations. The retrospective nature of the study may affect the outcome and in addition when conducting a long term follow-up the drop out of patients are commonly substantial. Limited sample size might also lead to Type II error and insufficient power to draw a definitive conclusion.

## Data availability

The data and methods used will be made available upon request.

## CRediT authorship contribution statement

**Åsa Edsander-Nord:** Methodology, Writing – review & editing. **Armin Assareh:** Investigation. **Martin Halle:** Methodology, Writing – review & editing. **Ann-Charlott Docherty Skogh:** Methodology, Writing – original draft, Writing – review & editing.

## Conflict of interest

The authors declare no financial interests or personal relationships that could have appeared to influence the work reported in this paper.
